# Spontaneous Adrenal Hemorrhage in Pregnancy: A Case Series

**DOI:** 10.1155/2017/3167273

**Published:** 2017-03-22

**Authors:** Ankita Gupta, Ruby Minhas, Hayley S. Quant

**Affiliations:** Department of Obstetrics and Gynecology, Crozer Chester Medical Center, One Medical Center Boulevard, Upland, PA 19013, USA

## Abstract

*Background*. Abdominal pain during pregnancy has a broad differential diagnosis which includes spontaneous adrenal hemorrhage (SAH). There is scant literature available on optimal mode of delivery in stable patients.* Cases*. Patient 1 was a 35-year-old nullipara who presented at 36 weeks of gestation with left flank pain. Patient 2 was a 27-year-old multipara at 38 weeks who presented with left upper quadrant pain. Diagnosis of SAH was made by CT scan and both were managed with pain control, serial hemoglobin assessments, and abdominal exams resulting in uncomplicated vaginal deliveries.* Conclusion*. SAH, although rare, is an important consideration when evaluating abdominal and flank pain in pregnancy. Management options vary from conservative management to surgical intervention depending on the stability of the patient.

## 1. Introduction

Abdominal pain, a common complaint during pregnancy, has a broad differential diagnosis which includes spontaneous adrenal hemorrhage (SAH). While autopsy reports reveal that between 0.03 and 1.8% of unselected cases demonstrate adrenal hemorrhage, the incidence among pregnant women is unknown [1]. A high index of suspicion is necessary to diagnose SAH as most patients present with nonspecific symptoms including abdominal or flank pain and fever [2, 3]. Rarely, patients can develop massive retroperitoneal bleeding and present with hemodynamic instability [1, 4, 5]. If bilateral, SAH can lead to adrenal crisis and shock necessitating emergency laparotomy and adrenalectomy [2, 5]. We present two cases of symptomatic SAH in the third trimester of pregnancy with successful conservative management.

## 2. Case Presentation

### 2.1. Case #1

A 35-year-old nulliparous patient at 36 weeks of gestation presented to labor and delivery complaining of sudden onset, left sided back pain which radiated anteriorly. She denied any other gastrointestinal or urinary symptoms. The patient had a past medical history significant only for chronic hypertension and a past surgical history of laparoscopic Roux en Y gastric bypass two years priorly. She denied any prior complications related to this surgical history. The pregnancy was further complicated by gestational diabetes class A1. On presentation the patient had a blood pressure of 162/80 and heart rate of 70 beats per minute and was saturating 99% on room air. Initial exam revealed the abdomen to be tender to palpation in left upper quadrant with no guarding and no peritoneal signs. There were no symptoms of labor and fetal status was reassuring.

**Figure 1:**
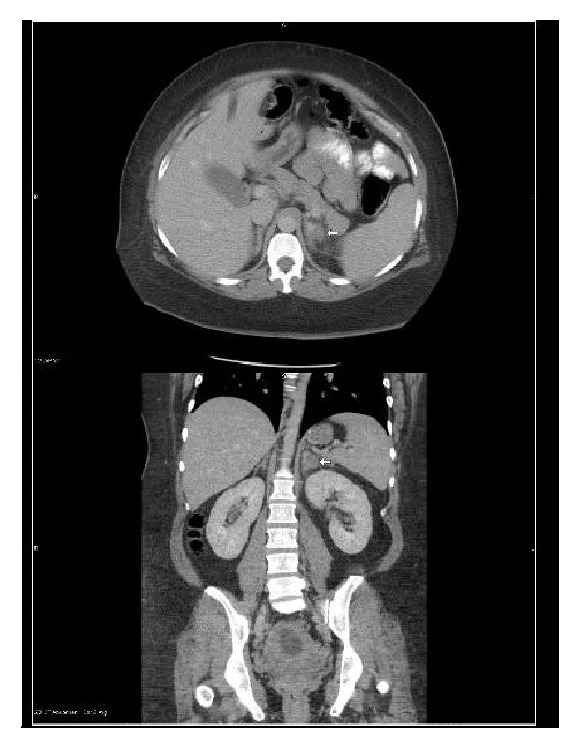
CT scan findings with arrow pointing at left adrenal hemorrhage.

Complete blood count, coagulation studies, liver function tests, amylase, and lipase were all within normal limits with a hemoglobin of 12.4 g/dL. A urinalysis demonstrated rare bacteria and calcium oxalate crystals, but no blood. A renal ultrasound showed no evidence of hydronephrosis, mass, or stone. An obstetrical ultrasound revealed a live singleton fetus with a normal appearing anterior placenta and appropriate fetal growth. Intravenous narcotics were required for adequate pain control. General surgery was consulted given her history of gastric bypass and concern for a possible related complication. A CT scan of the abdomen demonstrated a mildly enlarged left adrenal gland with areas of hyperdensity consistent with acute left adrenal hemorrhage (Figure 1). This was a new finding compared with a CT scan performed one year priorly. The patient denied any history of abdominal trauma or anticoagulation. She was admitted to the Surgical Intensive Care Unit where she was comanaged by Maternal Fetal Medicine and General Surgery. She was monitored with serial hemoglobin assessments and abdominal examinations and remained clinically and hemodynamically stable. She was discharged home at 37 weeks of gestation after a 4-day hospitalization and returned for induction of labor at 39 weeks of gestation due to chronic hypertension and gestational diabetes. The patient had an uncomplicated, spontaneous vaginal delivery of a female neonate weighing 7 pounds and 4 ounces with APGARs of 8 at 1 minute and 9 at 5 minutes. Her postpartum course was uncomplicated and interval imaging study to assess resolution of the adrenal hemorrhage was planned.

### 2.2. Case #2

**Figure 2:**
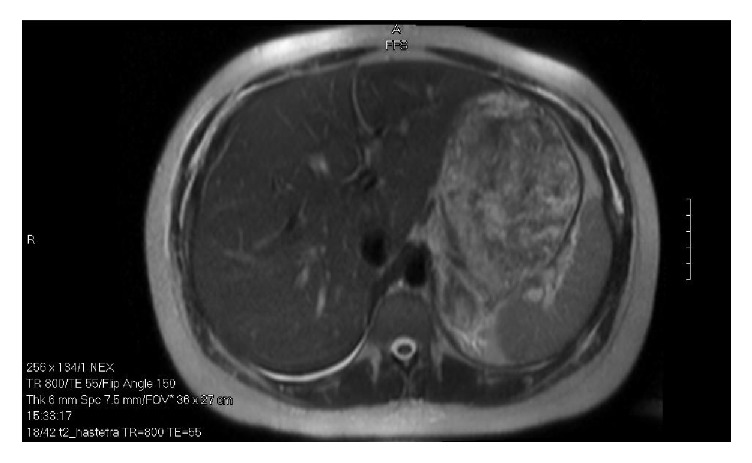
MRI findings of left adrenal hemorrhage.

A 27-year-old multiparous patient presented to labor and delivery at 38 weeks of gestation for evaluation of sudden onset, left upper quadrant pain that radiated to the midline. The patient also reported regular, painful contractions. She had a past medical history significant for nephrolithiasis during her previous pregnancy, bipolar disease, and migraines. Prior surgeries included a diagnostic laparoscopy for chronic pelvic pain, Loop Electrosurgical Excision Procedure, and 2 elective terminations of pregnancy. The patient was a 5-6-cigarette/day smoker. On admission vital signs were within normal limits. Physical examination revealed her abdomen to be soft and minimally tender to palpation. A complete blood count was within normal limits with a hemoglobin of 12.4 g/dL. A urine drug screen was negative and a urinalysis revealed no blood or evidence of infection. Fetal status was reassuring and an obstetric ultrasound revealed no apparent pathology. The patient's pain improved with intravenous narcotics, antacids, and a muscle relaxer and was attributed to a likely musculoskeletal etiology. However, the patient was found to be in spontaneous labor and progressed to deliver a male infant without complication. Intrapartum pain relief was provided via epidural. On the first postpartum day the patient again complained of left sided flank and upper abdominal pain and was presumptively treated for nephrolithiasis, given the clinical presentation and history, with intravenous narcotics and hydration. Despite treatment, the pain worsened and was associated with nausea and two episodes of emesis. A CT scan of the abdomen revealed enlargement and mild heterogeneity of the left adrenal gland with surrounding fat stranding consistent with acute left adrenal hemorrhage. An MRI performed at the request of the attending physician confirmed these findings as seen in Figure 2. She denied any trauma or use of anticoagulants. Total cortisol level was normal at 14.7 ug/dL with a low ACTH of 5.0 pg/mL. The patient was monitored with serial assessments of hemoglobin and remained clinically and hemodynamically stable. Her pain was controlled with intravenous narcotics. She was discharged on postpartum day 4 with a plan for short interval follow-up. A CT scan performed 8 months later revealed unremarkable adrenal glands with complete resolution of the previously identified hemorrhage.

## 3. Discussion

If unrecognized, adrenal hemorrhage can lead to adrenal crisis, shock, and theoretically death for both mother and fetus and should be considered in the differential diagnosis of abdominal pain in pregnancy [3, 4]. Presenting symptoms are similar to those in nonpregnant patients and include acute onset flank, abdominal or even chest pain, nausea, vomiting, or hypotension [4, 5]. In order to be classified as spontaneous and idiopathic there can be no history of trauma, anticoagulation, tumor, or sepsis [2, 4–6].

While the initial abdominal imaging study in pregnancy is typically ultrasound, sonographic findings of adrenal hemorrhage are nonspecific. MRI or CT scan is needed to confirm the diagnosis and to evaluate for potential underlying etiology such as pheochromocytoma or malignant tumor [2]. On MRI, adrenal hemorrhage appears as a heterogeneous mass with enlargement of one or both adrenal glands while a contrast CT scan demonstrates adrenal echogenicity, streaky appearance of the perirenal fat, perinephric hematoma, or a retroperitoneal hematoma in a massive bleed [2, 4]. A follow-up MRI or CT scan is usually recommended to confirm stability or resolution of the hematoma, especially if a conservative approach is adopted. Recommended laboratory evaluations include serial hemoglobin measurements as well as assessment of adrenal function. Work-up should also rule out disseminated intravascular coagulation and thrombotic thrombocytopenic purpura. Consideration should be given to inherited and acquired thrombophilias, including antiphospholipid syndrome, as potential etiologies of adrenal vein thrombosis and subsequent hemorrhage [3]. In both of the above reported cases platelet count and coagulation parameters were within normal limits and there was no clinical suspicion for thrombophilia.

Appropriate management of SAH in pregnancy depends on the stability of the patient. Conservative management includes supportive therapy with intravenous fluids, pain control, and serial hemoglobin assessments with blood transfusion and correction of coagulopathy as indicated [3]. Close monitoring of fetal status is warranted. Preterm delivery may be indicated if a patient is unstable, worsening, or if adrenal hemorrhage is associated with severe preeclampsia or eclampsia [2]. In hemodynamically unstable patients with ongoing hemorrhage arterial embolization can be considered, but severely ill patients may warrant emergent adrenalectomy [3, 7]. There is scant literature available on optimal mode of delivery but, in a stable patient, vaginal delivery can be safely undertaken as demonstrated in the reported cases.

## 4. Conclusion

SAH, although rare, is an important consideration when evaluating abdominal and flank pain in pregnancy. Diagnosis requires a high index of suspicion, particularly when more common etiologies of pain are excluded as was demonstrated in the two cases presented in this report. Diagnosis can be made by MRI or CT scan. In a clinically stable pregnant patient with SAH conservative management and vaginal delivery are safe and appropriate.
